# Comparative Analysis of Seven Equations for Estimated Glomerular Filtration Rate and Their Impact on Chronic Kidney Disease Categorization in Korean Patients at Local Clinics and Hospitals

**DOI:** 10.3390/jcm13071945

**Published:** 2024-03-27

**Authors:** Rihwa Choi, Sang Gon Lee, Eun Hee Lee

**Affiliations:** 1 Department of Laboratory Medicine, Green Cross Laboratories, Yongin 16924, Republic of Korea; pirate0720@naver.com; 2 Department of Laboratory Medicine and Genetics, Samsung Medical Center, Sungkyunkwan University School of Medicine, Seoul 06351, Republic of Korea; 3 Green Cross Laboratories, Yongin 16924, Republic of Korea

**Keywords:** glomerular filtration rate, chronic kidney disease, cystatin C, equation, Korea

## Abstract

**(1) Background:** Accurate estimation of the glomerular filtration rate (eGFR) is essential for the early detection of chronic kidney disease (CKD), targeted interventions, and ongoing monitoring. Although various equations for calculating eGFR exist, comparative studies on eGFR levels and the impact of these equations on CKD prevalence are limited in the Korean population. **(2) Methods:** We compared eGFR levels calculated using seven equations and investigated the prevalence of CKD through a retrospective analysis of the data from Korean adult patients who visited local clinics and hospitals and underwent simultaneous serum creatinine (Cr) and cystatin C (Cys-C) measurements. The equations analyzed were: 2006 MDRD, 2009 CKD-EPI Cr, 2012 CKD-EPI Cys-C, 2012 CKD-EPI Cr & Cys-C, 2021 CKD-EPI Cr, 2021 CKD-EPI Cr & Cys-C, and 2021 EKFC. **(3) Results:** This study included 6688 Korean patients (3736 men and 2952 women; median age: 61.4; IQR: 47.2–73.4). Among the equations, the median eGFR levels were the highest when using the 2021 CKD-EPI Cr & Cys-C equation (85.1 mL/min/1.73 m^2^) and the lowest when using the 2006 MDRD equation (73.4 mL/min/1.73 m^2^). The highest prevalence of decreased eGFR < 60 mL/min/1.73 m^2^ (equivalent to or worse than G3a CKD) was noted with the 2012 CKD-EPI Cys-C equation (32.4%), while the lowest was with the 2021 CKD-EPI Cr equation (22.9%), resulting in a maximum prevalence difference of 9.5%. **(4) Conclusions:** The prevalence of CKD varies based on the eGFR equation used and the patient’s age. Equations that include Cys-C may identify a larger number of patients with decreased kidney function.

## 1. Introduction

Chronic kidney disease (CKD) is a growing global health concern, and accurate estimation of the glomerular filtration rate (eGFR) is essential for early CKD detection, target-ed interventions, and ongoing monitoring [1]. The established criteria for CKD in adults include a glomerular filtration rate (GFR) below 60 mL/min/1.73 m^2^ for over 3 months [1,2,3,4]. Various equations utilizing serum levels of endogenous filtration markers have been developed and adopted in clinical settings to estimate the GFR [1]. These markers include serum creatinine (Cr), which is standardized and calibrated against isotope dilution mass spectrometry by the National Institute of Standards and Technology, and serum cystatin C (Cys-C), which is standardized through methods endorsed by the Institute for Reference Materials and Measurements of the International Federation of Clinical Chemistry and Laboratory Medicine (IFCC) and traceable to ERM-DA471/IFCC [5,6].

In Korea, the estimated glomerular filtration rate (eGFR) has commonly been calculated using the 2006 Modification of Diet in Renal Disease (2006 MDRD) Study Equation and the 2009 Chronic Kidney Disease Epidemiology Collaboration (CKD-EPI) creatinine equation (2009 CKD-EPI Cr), both of which are based on standardized serum creatinine (Cr) levels, age, sex, and race [7,8]. In 2012, the Kidney Disease: Improving Global Outcomes (KDIGO) clinical practice guidelines recommended the use of serum cystatin C (Cys-C)—which is less influenced by age, race, and muscle mass compared to serum Cr—for the calculation of the eGFR, either with Cys-C alone (2012 CKD-EPI Cys-C) or in combination with Cr (2012 CKD-EPI Cr & Cys-C) [1,9]. However, these equations used Cys-C measurements that were not standardized with ERM-DA471/IFCC [1,9]. In 2021, the CKD-EPI introduced new equations that omitted race as a variable: 2021 CKD-EPI Cr and 2021 CKD-EPI Cr & Cys-C, based on Cys-C test results using standardized analytical methods traceable to ERM-DA471/IFCC [5]. Additionally, the new European Kidney Function Consortium (EKFC) introduced the 2021 EKFC equation based on serum Cr, age, and sex [10].

In Korea, the Korean Academy of Medical Sciences and the Korea Disease Control and Prevention Agency (KDCA) have published an evidence-based guideline for chronic kidney disease in primary care, advising cautious use of the new eGFR equations due to their recent development [3]. The guideline highlights that the performance of these equations, with or without race as a variable, may vary across different ethnic groups and clinical settings [3,5,11,12,13,14]. Among the seven equations, the Korean Society of Nephrology recommends the 2009 CKD-EPI creatinine equation for evaluating CKD. In contrast, the National Health Insurance Service (NHIS) of Korea had been utilizing the 2006 MDRD equation for CKD screening when the eGFR was less than 60 mL/min/1.73 m^2^ or serum creatinine exceeded 1.5 mg/dL. However, the NHIS changed and began recommending the use of the 2009 CKD-EPI creatinine equation starting from 1 January 2024 [3,15,16]. Additionally, due to the Cys-C assay being more than three times more expensive in Korea, prior studies on eGFR typically involved patients from university hospitals or participants in national cohort studies [12,13,14,17]. According to data from the Health Insurance Review and Assessment Service (HIRA) of Korea, 53,599,947 Cr tests were conducted in 2022, with about 37.3% of the total testing costs incurred by local clinics and hospitals, whereas 335,412 Cys-C tests were performed in the same year, with approximately 14.3% of the total Cys-C testing costs being attributable to these local healthcare settings [16,18]. Therefore, data on the use of Cys-C and the performance of various eGFR equations utilizing Cys-C in patients visiting local clinics and hospitals remain scarce in Korea.

As Green Cross Laboratories is one of the largest referral laboratories in Korea offering clinical analysis services for both serum Cr and Cys-C, as requested by local clinics and hospitals nationwide, the aim of this study was to conduct a comparative analysis of eGFR using various equations, including those utilizing Cr and Cys-C, in a large adult Korean population visiting local healthcare facilities. This investigation aimed to assess the impact of different eGFR calculation equations on the prevalence of decreased eGFR as a diagnostic tool for evaluating CKD in the adult Korean population.

## 2. Materials and Methods

### 2.1. Study Subjects

We conducted a retrospective review of data from the laboratory information system of Green Cross Laboratories, covering the period from 1 January 2022 to 31 December 2023, for Korean adults (aged > 20 years) who visited local clinics and hospitals for serum Cr and Cys-C testing. Data entries with missing age and sex information were excluded. To facilitate the comparison of seven equations, we also excluded results that only had Cr or only Cys-C values. Given the study’s objective to assess the prevalence of decreased eGFR, which is indicative of CKD in the Korean population, we further excluded duplicate entries from the same individuals.

### 2.2. Analytical Methods

Serum Cr levels were measured using the Creatinine Jaffe Gen.2 kits (CREJ2, Roche, Mannheim, Germany) via a kinetic colorimetric assay on c702 chemistry analyzers (Roche, Mannheim, Germany). Serum Cys-C levels were determined using Cias Cys-C reagent kits (Kanto Chemical, Tokyo, Japan) on an AU680 analyzer (Beckman Coulter, Tokyo, Japan). The analytical methods were consistently maintained and remained unchanged throughout the study period. The accuracy of the serum Cr and Cys-C measurements was verified through participation in proficiency testing programs, including the CYS survey by the College of American Pathologists [19] and both the accuracy-based creatinine analysis and the cystatin C survey by the Korean Association of External Quality Assessment Service (KEQAS) [20].

### 2.3. Definitions

eGFR levels and the prevalence of decreased eGFR < 60 mL/min/1.73 m^2^ (equivalent to or worse than G3a CKD) were compared across seven distinct equations: (1) 2006 MDRD [7], (2) 2009 CKD-EPI Cr [8], (3) 2012 CKD-EPI Cys-C [1,9], (4) 2012 CKD-EPI Cr & Cys-C [1,9], (5) 2021 CKD-EPI Cr [5], and (6) 2021 CKD-EPI Cr & Cys-C [5], and (7) 2021 EKFC [10].

The classification of decreased kidney function and the CKD groups based on eGFR followed the current clinical guidelines: G1 (normal or high eGFR, ≥90 mL/min/1.73 m^2^); G2 (mildly decreased eGFR, 60–89 mL/min/1.73 m^2^); G3a (mildly to moderately decreased eGFR, 45–59 mL/min/1.73 m^2^); G3b (moderately to severely decreased eGFR, 30–44 mL/min/1.73 m^2^); G4 (severely decreased eGFR, 15–29 mL/min/1.73 m^2^); and G5 (kidney failure, <15 mL/min/1.73 m^2^) [1,3].

### 2.4. Statistical Analysis

The descriptive statistics are presented as the median and interquartile range (IQR) for non-normally distributed quantitative data. Linear regression analysis was conducted to evaluate the association between eGFR and increasing age. The quantitative comparison of eGFR levels derived from each equation was carried out using the ANOVA test. The qualitative assessment of the prevalence of decreased eGFR across CKD stages (G1 to G5) was conducted using the chi-squared test, with stratification by age group. Agreement among the CKD classification grades (G1 to G5), based on each of the seven equations, was investigated. Analyses of overall agreement in defining decreased eGFR (CKD grade) using each equation, in comparison to the 2006 MDRD equation and the 2009 CKD-EPI Cr equation as reference methods, were conducted.

Given that eGFR equations have not been widely validated for subjects aged over 75 years, a subgroup analysis was conducted on subjects aged 20 to 75 years, after excluding those older than 75 years [1,21].

A *p*-value of less than 0.05 was deemed statistically significant. All analyses were performed using the MedCalc statistical software version 20.216 (MedCalc Software Ltd., Ostend, Belgium).

## 3. Results

### 3.1. Baseline Characteristics of Study Subjects

Over the two-year study period, 6688 Korean patients (median age: 61.4 years, IQR: 47.2–73.4; 3736 men and 2952 women) underwent simultaneous serum Cr and Cys-C testing. Approximately 69.4% (4644) of the subjects had their serum Cr and Cys-C measured only once during the study, while the remaining 30.6% (2044/6688) had measurements taken two or more times. For the analysis of decreased eGFR prevalence, only the first measurements from each individual were considered, excluding the subsequent repeats. The baseline characteristics of the study subjects are summarized in Table 1.

Among the seven eGFR equations, the median eGFR levels were highest with the 2021 CKD-EPI Cr & Cys-C equation (85.1 mL/min/1.73 m^2^), followed, in descending order, by the 2021 CKD-EPI Cr, 2009 CKD-EPI Cr, 2012 CKD-EPI Cr & Cys-C, 2012 CKD-EPI Cys-C, and 2006 MDRD equations (73.4 mL/min/1.73 m^2^). Significant differences in the eGFR values were observed among the equations (*p* < 0.05). The 2012 CKD-EPI Cys-C equation revealed the highest prevalence of decreased eGFR < 60 mL/min/1.73 m^2^ (equivalent to or worse than G3a), with 32.4%, while the 2021 CKD-EPI Cr equation showed the lowest with 22.9%, yielding a maximum prevalence difference of 9.5%.

### 3.2. eGFR Levels and CKD Classification by Age

**Figure 1:**
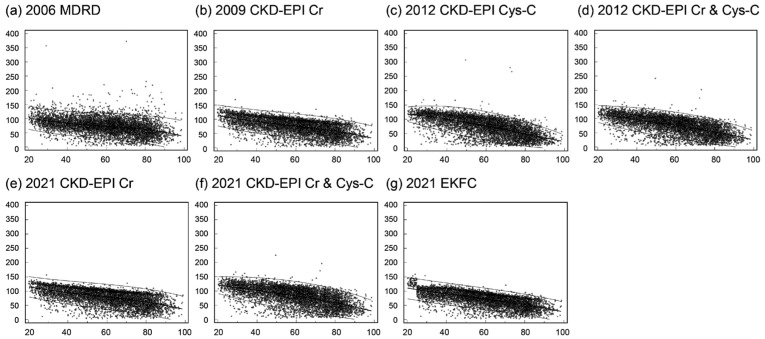
Estimated glomerular filtration rate (eGFR) by age: (**a**) using the 2006 MDRD equation, (**b**) using the 2009 CKD-EPI Cr equation, (**c**) using the 2012 CKD-EPI Cys-C equation, (**d**) using the 2012 CKD-EPI Cr & Cys-C equation, (**e**) using the 2021 CKD-EPI Cr equation, (**f**) using the 2021 CKD-EPI Cr & Cys-C equation, and (**g**) using the 2021 EKFC equation. The bold median line indicates the median, and the thin lines represent the upper and lower limits of the central 95th percentiles (2.5th and 97.5th percentiles).

The eGFR values calculated with each equation and stratified by age are depicted in Figure 1. The eGFR values determined using the seven equations showed a gradual decline with increasing age. The 2006 MDRD equation exhibited a broader distribution of eGFR values, with some exceeding 150 mL/min/1.73 m^2^, whereas the 2009 CKD-EPI Cr, 2021 CKD-EPI Cr, and 2021 EKFC equations demonstrated a narrower distribution of eGFR values.

The correlation coefficient (*r*) was highest for the eGFR values calculated using the 2012 CKD-EPI Cys-C equation (*r* = 0.66, *p* < 0.001), followed by the 2021 EKFC equation (*r* = 0.65, *p* < 0.001) and the 2012 CKD-EPI Cr & Cys-C equation (*r* = 0.63, *p* < 0.001), with the lowest correlation observed for the eGFR based on the 2006 MDRD equation (*r* = 0.38, *p* < 0.001).

**Figure 2:**
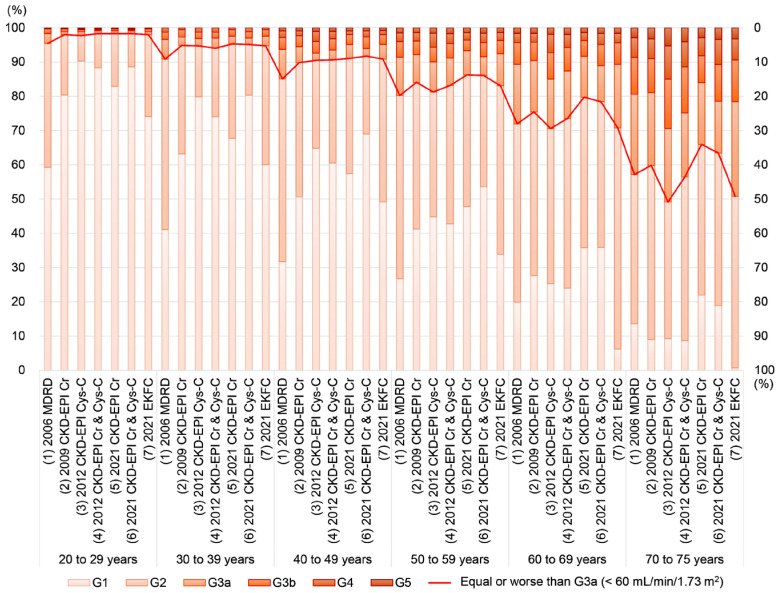
Prevalence of decreased eGFR by CKD grade classification and age based on each equation. The left *y*-axis represents the prevalence of each CKD grade (G1, G2, G3a, G3b, G4, and G5). The prevalence of decreased eGFR < 60 mL/min/1.73 m^2^ (equivalent to or worse than G3a) is depicted as a red line, with the corresponding *y*-axis on the right.

The prevalence of decreased eGFR by CKD grade classification across different age groups is summarized in Figure 2. The prevalence of decreased eGFR increases with age. Among the seven equations, the 2006 MDRD equation indicated the highest prevalence of eGFR values of less than 60 mL/min/1.73 m^2^ (equivalent to or worse than G3a) in the subjects under 60 years of age. However, for the subjects aged 60 years and above, the 2012 CKD-EPI Cys-C equation showed the highest prevalence of decreased eGFR below 60 mL/min/1.73 m^2^. In the age group comprising subjects aged 70 years and older, the 2021 CKD-EPI Cr equation demonstrated the lowest prevalence of decreased eGFR (<60 mL/min/1.73 m^2^).

### 3.3. Comparison of eGFR Levels and CKD Classification

**Figure 3:**
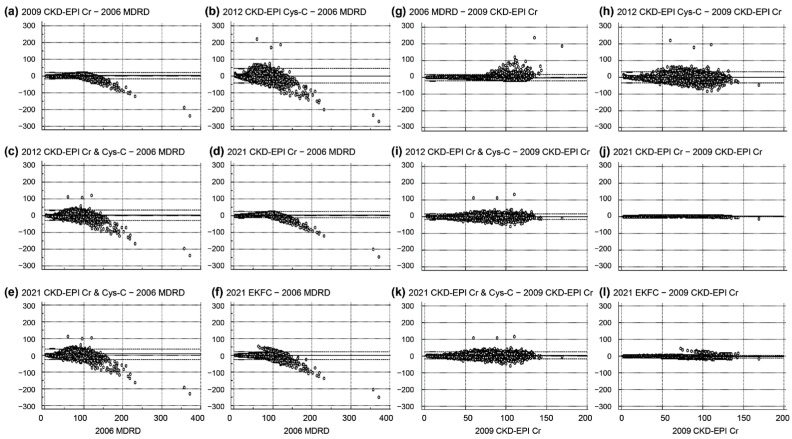
Bland–Altman plot illustrating differences in eGFR levels. The *y*-axis represents the differences in eGFR levels (mL/min/1.73 m^2^) between the 2006 MDRD equation and each of the other equations (panels (**a**–**f**); each equation minus 2006 MDRD) and between the 2009 CKD-EPI Cr equation and each of the other equations (panels (**g**–**l**); each equation minus 2009 CKD-EPI Cr). The *x*-axis for panels (**a**–**f**) spans from 0 to 400 mL/min/1.73 m^2^ based on the 2006 MDRD equation, and for panels (**g**–**l**), it ranges from 0 to 200 mL/min/1.73 m^2^ based on the 2009 CKD-EPI Cr equation.

Given that the 2006 MDRD equation has been widely used for health checkups, following the guidelines of the NHIS, Korea, we investigated the comparison of the eGFR values derived from each equation with those from the 2006 MDRD equation. Additionally, since the 2009 CKD-EPI Cr equation is recommended by the Korean Society of Nephrology’s clinical practice guidelines, we also examined how the eGFR values from each equation compared to those from the 2009 CKD-EPI Cr. Bland–Altman plots illustrating the differences between the 2006 MDRD equation and the others, as well as between the 2009 CKD-EPI Cr equation and the others, are summarized in Figure 3. The Bland–Altman plot comparing the 2006 MDRD equation with the others (Figure 3a–f) shows the smallest difference with the 2009 CKD-EPI Cr equation (median absolute percentage error of 6.7%). Similarly, the Bland–Altman plot comparing the 2009 CKD-EPI Cr with the other equations (Figure 3g–l) demonstrates the smallest difference with the 2021 CKD-EPI Cr equation (median absolute percentage error of 5.2%).

**Figure 4:**
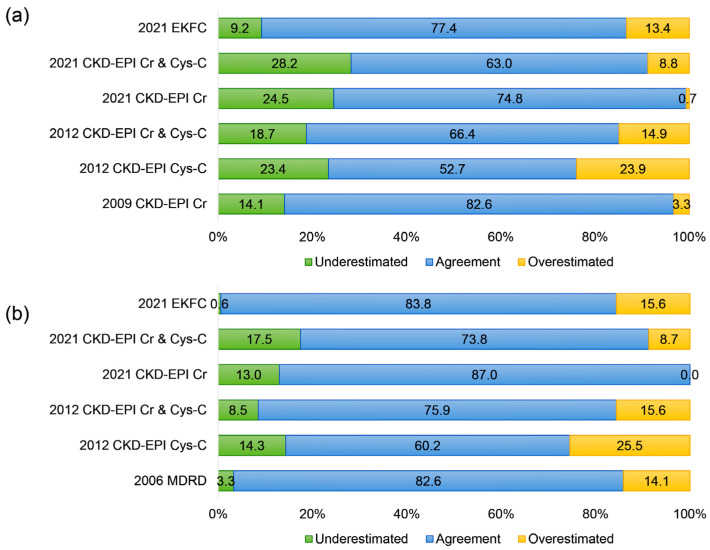
Overall agreement in defining CKD grade classification: (**a**) using the 2006 MDRD equation and (**b**) using the 2009 CKD-EPI Cr equation. “Underestimation” in (**a**) indicates cases where the CKD grade determined with the 2006 MDRD equation is greater than the CKD grade determined by each of the other equations, and in (**b**), it indicates cases where the CKD grade determined with the 2009 CKD-EPI Cr equation is greater than the CKD grade determined by each of the other equations. “Overestimation” in (**a**) signifies cases where the CKD grade determined with the 2006 MDRD equation is less than the CKD grade determined by each of the other equations, and in (**b**), it signifies cases where the CKD grade determined with the 2009 CKD-EPI Cr equation is less than the CKD grade determined by each of the other equations.

An analysis of the overall agreement in defining decreased eGFR (CKD grade) using each equation, in comparison with the 2006 MDRD equation and the 2009 CKD-EPI Cr equation as reference methods, was performed and is summarized in Figure 4. Compared to the 2006 MDRD equation (Figure 4a), the 2009 CKD-EPI Cr equation exhibited the highest agreement in the CKD grading (82.6%), while the 2012 CKD-EPI Cys-C equation showed the lowest agreement (52.7%) and more frequently overestimated the CKD grades than the 2006 MDRD (23.9%). Among the other equations, the 2021 CKD-EPI Cr & Cys-C equation had the highest proportion of underestimated CKD grades (28.2%) compared to the 2006 MDRD. Excluding the 2006 MDRD equation, the 2021 EKFC equation had the lowest proportion of underestimation (9.2%) compared to the 2006 MDRD.

When compared to the 2009 CKD-EPI Cr equation (Figure 4b), the 2021 CKD-EPI Cr equation showed the highest agreement in the CKD grading (87.0%), while the 2012 CKD-EPI Cys-C equation demonstrated the lowest agreement (60.2%) and more frequently overestimated the CKD grades than the 2009 CKD-EPI Cr equation (25.5%). Among the other equations, the 2021 CKD-EPI Cr equation did not overestimate the CKD grades in any patients. Excluding the 2009 CKD-EPI Cr equation, the 2021 EKFC equation had the lowest proportion of underestimation (9.2%) compared to the 2009 CKD-EPI Cr.

After excluding the subjects aged over 75 years, a subgroup analysis was conducted with data from 5229 subjects, as presented in Supplementary Table S1 and Figures S1–S3. The equations that yielded the highest and lowest eGFR levels (the 2021 CKD-EPI Cr & Cys-C and the 2006 MDRD, respectively) in this subgroup analysis, which included subjects aged 20 to 75 years, remained consistent with those observed in the entire study population. Although the equation that showed the lowest prevalence of decreased eGFR (<60 mL/min/1.73 m^2^; the 2021 CKD-EPI Cr) in the subgroup analysis was consistent with that in the full dataset, the equation associated with the highest prevalence of decreased eGFR differed (the 2006 MDRD in the subgroup vs. the 2012 CKD-EPI Cys-C in all subjects). However, the prevalence of decreased eGFR (classified according to CKD grade) across the age groups was similar between the entire study cohort (*n* = 6688) and the subgroups (*n* = 5229), as shown in Figure 2 and Figure 3 and Supplementary Figures S1–S3.

## 4. Discussion

In this study, we explored the variances in eGFR calculated using seven equations and the prevalence of CKD grades associated with decreased eGFR among the adult Korean population visiting local clinics and hospitals. We conducted retrospective data analysis of the results from simultaneous serum Cr and Cys-C measurements to compare the seven eGFR equations. Given that the latest equations were introduced in 2021, there are limited Korean studies employing various equations for the calculation of eGFR (refer to Table 2). A key strength of this study is its inclusion of a large cohort of adult Korean individuals from local clinics and hospitals across Korea, alongside the utilization of multiple equations for eGFR comparison [13,22,23,24,25,26,27].

In this study, we confirm previous findings showing that eGFR decreases with age and that the proportion of decreased eGFR increases with age, underscoring the need for age-specific thresholds for CKD [2,13,22,23,24,25,26,27]. The 2006 MDRD equation showed the lowest correlation coefficient with age in this study, aligning with previous studies conducted in healthy subjects [23]. The ranking from the highest to the lowest of the median (or mean) eGFR levels calculated from each equation is consistent with prior findings reported in the Korean population under various clinical settings, in other ethnic groups (non-black individuals) and in the Korea National Health and Nutrition Examination Survey 2019 data [5,11,13,22,23,24,25,26,27]. Notably, the 2006 MDRD and 2021 EKFC equations yielded the lowest eGFR levels compared to the other equations, potentially leading to an underestimation of CKD grades in the Korean adult population [13,22,23,24,25,26,27].

The 2006 MDRD equation, developed for individuals with CKD, has significant limitations, including imprecision and a systematic underestimation (bias) of measured GFR at higher levels [1,28]. Consequently, when a 2006 MDRD eGFR value of ≥60 mL/min/1.73 m^2^ is obtained, it is reported as “≥60 mL/min/1.73 m^2^” rather than as its absolute value, in accordance with the National Kidney Disease Education Program (NKDEP) recommendation [1,28]. Given this limitation, the 2006 MDRD equation may not be the best choice for screening in the general population, particularly because of its less accurate estimations at GFR levels ≥ 60 mL/min/1.73 m^2^ [1,3]. In contrast, the serum Cr-based CKD-EPI equation generally offers more accurate estimations at GFR levels ≥ 60 mL/min/1.73 m^2^, and the Korean Society of Nephrology also recommends the 2009 CKD-EPI creatinine equation for CKD evaluation [1,3]. The NHIS in Korea recently revised its recommendations, shifting from the 2006 MDRD equation to the 2009 CKD-EPI creatinine equation, in conjunction with the serum creatinine level criterion (>1.5 mg/dL). Future studies should monitor changes in the prevalence of CKD in Korea and take into account the equations that are used.

Meanwhile, the qualitative comparison of the CKD category agreement (G1 to G5) between the 2006 MDRD and 2009 CKD-EPI Cr equations, the 2021 EKFC and 2006 MDRD equations, and the 2021 EKFC and 2009 CKD-EPI Cr equations is in line with the results from the other equations in this study (Figure 2 and Figure 4). Although the eGFR levels calculated from the 2009 CKD-EPI Cr and 2021 CKD-EPI Cr equations appear comparable (Figure 3j), the agreement in CKD categories shows a higher proportion of underestimation compared to the 2009 CKD-EPI Cr equation. These findings align with previous recommendations regarding the cautious use of newly developed equations in Korean subjects, as the 2009 CKD-EPI Cr and 2012 CKD-EPI Cr & Cys-C equations demonstrated better performance in the Korean population [3,13,25]. This suggests that, while quantitative eGFR level differences were observed, the clinical impact on qualitative CKD categorization may differ from the impact on the quantitative levels [9,13,29].

Furthermore, this study found that the highest prevalence of decreased eGFR (equal to or worse than G3a) was observed with the 2006 MDRD equation in the subjects aged under 60 years and with the 2012 CKD-EPI Cys-C equation in the subjects aged 60 years and over, indicating that the accuracy and performance of each equation may vary between younger and older populations [2,30]. In the subjects aged 70 years and over, the 2021 CKD-EPI Cr equation showed the lowest prevalence of decreased eGFR and the largest proportion of underestimated CKD grades compared to the 2006 MDRD and 2009 CKD-EPI Cr equations. This finding suggests that Cys-C, which is known to be less affected by muscle mass than creatinine, and the use of Cys-C-derived equations could be valuable in the elderly population [30,31]. In this study, with a median age of 61.0 years, the subjects underwent simultaneous serum Cr and Cys-C testing, indicating that Korean physicians in local clinics and hospitals might use cystatin C tests in addition to creatinine, especially in elderly patients, given the characteristics of Cys-C [3]. The two cystatin C-derived equations (2012 CKD-EPI Cys-C and 2012 CKD-EPI Cr & Cys-C) gave the largest proportion of overestimated CKD categories compared to the 2006 MDRD and 2009 CKD-EPI Cr equations, with a maximum difference in the prevalence of decreased eGFR of 9.5%. This suggests that using the 2012 CKD-EPI Cys-C and 2012 CKD-EPI Cr & Cys-C equations could identify more patients as potential CKD cases. In the subgroup analysis of the current study, which included subjects aged 20 to 75 years, the patterns among the equations across different age groups were consistent with those from the subjects as a whole. Given that eGFR equations have not been widely validated for subjects aged over 75 years, future studies are needed to further explore the clinical implications and roles of each equation across different age groups and ethnic populations [1,2,28,31]. Factors associated with Cr generation, such as race, ethnicity, muscle mass, nutritional status, and the use of drugs like trimethoprim, cimetidine, and fenofibrate, as well as the presence of spectral and chemical substances that interfere with Cr assays in the specimen (bilirubin, glucose, ketones, and drugs), may influence the accuracy of Cr-based equations [1,4,5]. Similarly, factors associated with Cys-C measurements, including non-steady state conditions of the specimen, influences on Cys-C generation such as race, ethnicity, thyroid function, corticosteroid administration, diabetes, obesity, and the presence of interfering substances in the specimen like heterophilic antibodies, may impact the reliability of Cys-C-based equations [1,4,5].

The limitations of the present study include the lack of clinical information associated with CKD and decreased eGFR, such as body mass index, comorbidities, and medications [1,4,31]. The aim of the current study was to evaluate eGFR values derived from various equations and the seroprevalence of different CKD grades among them. Consequently, clinical details pertaining to confirmatory tests, such as whether they entailed at least two measurements taken three months apart or more, were not evaluated. However, because standardized serum Cr and Cys-C measurements were used to calculate seven eGFR equations, and this approach is usually performed in clinical practice to assess subjects with CKD or decreased eGFR, the present study, which involved a large number of adult Koreans visiting local clinics and hospitals, may provide valuable insights into the comparative disease burden of CKD in adult Koreans.

## 5. Conclusions

In conclusion, this study compared seven equations for the calculation of eGFR (2006 MDRD, 2009 CKD-EPI Cr, 2012 CKD-EPI Cys-C, 2012 CKD-EPI Cr & Cys-C, 2021 CKD-EPI Cr, 2021 CKD-EPI Cr & Cys-C, and 2021 EKFC equations) and investigated their effect when assessing the prevalence of possible CKD in a large number of adult Koreans visiting local clinics and hospitals. The eGFR decreased with increasing age, confirming previous findings, and the prevalence of decreased eGFR (<60 mL/min/1.73 m^2^) varied among the seven equations in different age groups. The agreement of the CKD category based on the eGFR levels calculated from each equation varied compared to those calculated using the 2006 MDRD and 2009 CKD-EPI Cr equations, which are widely used in Korea. The prevalence of CKD using eGFR based on the 2012 CKD-EPI Cys-C and 2012 CKD-EPI Cr & Cys-C equations may result in increased seroprevalence. Future studies on the clinical impact of different equations are needed.

## Figures and Tables

**Table 1:** Baseline characteristics of 6688 study subjects.

Characteristics	Data
Age, year (median, IQR)	61.4 (47.2 to 73.4)
Age group (*n*, %)	
20 to 29 years	351 (5.2)
30 to 39 years	701 (10.5)
40 to 49 years	907 (13.6)
50 to 59 years	1200 (17.9)
60 to 69 years	1482 (17.9)
70 to 79 years	1177 (17.6)
≥80 years	870 (13.0%)
Sex (*n*, %)	
Men	3736 (55.9)
Women	2952 (44.1)
Serum Cr (mg/dL, median, IQR)	0.96 (0.77 to 1.20)
Serum Cys-C (mg/L, median, IQR)	0.98 (0.81 to 1.31)
eGFR using 2006 MDRD (mL/min/1.73 m^2^, median, IQR)	73.4 (56.8 to 89.7)
eGFR using 2009 CKD-EPI Cr (mL/min/1.73 m^2^, median, IQR)	79.0 (58.9 to 94.7)
eGFR using 2012 CKD-EPI Cys-C (mL/min/1.73 m^2^, median, IQR)	77.4 (51.0 to 101.4)
eGFR using 2012 CKD-EPI Cr & Cys-C (mL/min/1.73 m^2^, median, IQR)	79.0 (55.2 to 97.9)
eGFR using 2021 CKD-EPI Cr (mL/min/1.73 m^2^, median, IQR)	83.4 (62.5 to 98.8)
eGFR using 2021 CKD-EPI Cr & Cys-C (mL/min/1.73 m^2^, median, IQR)	85.1 (59.8 to 105.1)
eGFR using 2021 EKFC (mL/min/1.73 m^2^, median, IQR)	74.3 (55.1 to 89.6)
Decreased eGFR (<60 mL/min/1.73 m^2^, CKD ≥ G3a)	
Based on 2006 MDRD 2006 (*n*, %)	1926 (28.8%)
Based on 2009 CKD-EPI Cr (*n*, %)	1754 (26.2%)
Based on 2012 CKD-EPI Cys-C (*n*, %)	2175 (32.4%)
Based on 2012 CKD-EPI Cr & Cys-C (*n*, %)	1963 (29.3%)
Based on 2021 CKD-EPI Cr (*n*, %)	1536 (22.9%)
Based on 2021 CKD-EPI Cr & Cys-C (*n*, %)	1687 (25.1%)
Based on 2021 EKFC (*n*, %)	2028 (30.3%)

Abbreviations: Cr, creatinine; Cys-C, cystatin C; eGFR, estimated glomerular filtration rate.

**Table 2:** Studies comparing different equations for estimated glomerular filtration rate (eGFR) in the adult Korean population.

Ref.	Study Period	N of Subjects	Age (y)	Clinical Setting	No. of Eq	Equations	Analytical Methods for Cr	Analytical Methods for Cys-C	Findings (eGFR Levels)
This study	2022 to 2023	6688	61.4 **^a^**	Subjects underwent serum Cr and Cys-C measurement simultaneously, local clinics and hospitals	7	2006 MDRD, 2009 CKD-EPI Cr, 2012 CKD-EPI Cys-C, 2012 CKD-EPI Cr & Cys-C, 2021 CKD-EPI Cr, 2021 CKD-EPI Cr & Cys-C, and 2021 EKFC	Jaffe method, cobas 8000 system (Roche)	Cias Cys-C kit (Kanto Chemicals) on AU680 analyzer (Beckman Coulter)	2006 MDRD < 2021 EKFC < 2012 CKD-EPI Cys < 2012 CKD-EPI Cr & Cys-C < 2009 CKD-EPI Cr < 2021 CKD-EPI Cr & Cys-C
Choi et al. [23]	2019 to 2021	442,566	50.1	Health checkup, local clinics and hospitals	3	2006 MDRD, 2009 CKD-EPI Cr, and 2021 CKD-EPI Cr	Jaffe method, cobas 8000 system (Roche)	Cys-C was not included	2006 MDRD < 2009 CKD-EPI < 2021 CKD-EPI Cr
Kim et al. [24]	2018 to 2020	106,021	48.0 **^a^**	Annual physical checkup, single center	4	2009 CKD-EPI Cr, 2012 CKD-EPI Cr & Cys-C, 2021 CKD-EPI Cr & Cys-C, and 2021 EKFC	Jaffe method, Creatinine FS (DiaSys, Holzheim, Germany) on TBA-FX8 (Canon Medical Systems Corporation, Tokyo, Japan)	Gentian Cystatin C Immunoassay kit (Gentian Diagnostics AS Moss, Norway) on TBA-FX8 (Canon Medical Systems Corporation)	2021 EKFC < 2009 CKD-EPI Cr = 2012 CKD-EPI Cr & Cys-C < 2021 CKD-EPI Cr & Cys-C
Cho et al. [25]	2011 to 2021	187,139	58.2	AKI, single center (27,447 AKI patients and 159,692 controls)	3	2009 CKD-EPI Cr, 2021 CKD-EPI Cr, and 2021 EKFC	Not described	Cys-C was not included	2021 EKFC < 2009 CKD-EPI Cr < 2021 CKD-EPI Cr
Cho et al. [13]	2008 to 2013	239	49.4	Single center (201 CKD patients and 38 health volunteers)	5	2009 CKD-EPI Cr, 2012 CKD-EPI Cr & Cys-C, 2012 CKD-EPI Cys-C, 2021 CKD-EPI Cr, and 2021 CKD-EPI Cr & Cys-C	Jaffe method, Hitachi 7600 analyzer (200FR, Toshiba)	Particle-enhanced immunoturbidimetric assay, cobas 6000 system (Roche)	2009 CKD-EPI Cr < 2021 CKD-EPI Cr < 2012 CKD-EPI Cr & Cys-C < 2021 CKD-EPI Cr & Cys-C < 2012 CKD-EPI Cys-C
Jeong et al. [22]	2009 to 2020	1654	61.0 **^a^**	Subjects underwent ^51^Cr-EDTA GFR test, single center	3	2009 CKD-EPI Cr, 2012 CKD-EPI Cr, and 2021 EKFC	Jaffe method (Roche)	Cys-C was not included	2021 EKFC < 2009 CKD-EPI Cr < 2021 CKD-EPI Cr
Kim et al. [26]	2011 to 2015	2149	53.8	KNOW-CKD cohort	4	2009 CKD-EPI Cr, 2012 CKD-EPI Cr & Cys-C, 2021 CKD-EPI Cr, and 2021 CKD-EPI Cr & Cys-C	Jaffe method, ADVIA Chemistry XPT System (Siemens)	Particle-enhanced nephelometric immunoassay, BN II System (Siemens)	2012 CKD-EPI Cr & Cys-C < 2009 CKD-EPI Cr < 2021 CKD-EPI Cr & Cys-C < 2021 CKD-EPI Cr
Kim et al. [27]	2011 to 2015	2207	55.0 **^a^**	KNOW-CKD cohort	3	2009 CKD-EPI Cr, 2021 CKD-EPI Cr, and 2021 CKD-EPI Cr & Cys-C	Not described	Not described	2009 CKD-EPI Cr < 2021 CKD-EPI Cr < 2021 CKD-EPI Cr & Cys-C equations

Abbreviations: AKI, acute kidney injury; CKD, chronic kidney disease; Cr, creatinine; ^51^Cr-EDTA, chromium-51-ethylenediamine tetraacetic acid; Cys-C, cystatin-C; eq, equation; KNOW-CKD, KoreaN cohort study for Outcome in patients With Chronic Kidney Disease from eight tertiary hospitals without kidney replacement therapy; No., number; Ref., reference; y, years. Age presented as the mean. **^a^** Median age.

## Data Availability

The datasets generated and analyzed during the current study are available from the corresponding authors upon reasonable request.

## Supplementary Materials

The following supporting information can be downloaded at: https://www.mdpi.com/article/10.3390/jcm13071945/s1, Table S1: Baseline characteristics of study subjects aged 20 to 75 years (*n* = 5229), Figure S1: Prevalence of decreased eGFR by CKD grade classification and age based on each equation in subjects aged 20 to 75 years (*n* = 5229). The left *y*-axis represents the prevalence of each CKD grade (G1, G2, G3a, G3b, G4, and G5). The prevalence of decreased eGFR < 60 mL/min/1.73 m^2^ (equivalent to or worse than G3a) is depicted as a red line, with the corresponding *y*-axis on the right, Figure S2: Bland–Altman plot illustrating differences in eGFR levels in subjects aged 20 to 75 years. The *y*-axis represents the differences in eGFR levels (mL/min/1.73 m^2^) between the 2006 MDRD equation and each of the other equations (panels a to f; each equation minus 2006 MDRD) and between the 2009 CKD-EPI Cr equation and each of the other equations (panels g to l; each equation minus 2009 CKD-EPI Cr). The *x*-axis for panels (a–f) spans from 0 to 400 mL/min/1.73 m^2^ based on the 2006 MDRD equation, and for panels (g–l), it ranges from 0 to 200 mL/min/1.73 m^2^ based on the 2009 CKD-EPI Cr equation, Figure S3: Overall agreement in defining CKD grade classification in subjects aged 20 to 75 years: (a) using the 2006 MDRD equation and (b) using the 2009 CKD-EPI Cr equation. “Underestimation” in (a) indicates cases where the CKD grade determined with the 2006 MDRD equation is greater than the CKD grade determined by each of the other equations, and in (b), it indicates cases where the CKD grade determined with the 2009 CKD-EPI Cr equation is greater than the CKD grade determined by each of the other equations. “Overestimation” in (a) signifies cases where the CKD grade determined with the 2006 MDRD equation is less than the CKD grade determined by each of the other equations, and in (b), it signifies cases where the CKD grade determined with the 2009 CKD-EPI Cr equation is less than the CKD grade determined by each of the other equations.
