# Supplementary material for: Comparative Analysis of Seven Equations for Estimated Glomerular Filtration Rate and Their Impact on Chronic Kidney Disease Categorization in Korean Patients at Local Clinics and Hospitals
Source: J Clin Med. 2024 Mar 27;13(7):1945. doi: 10.3390/jcm13071945 (PMC11012467; doi:10.3390/jcm13071945)
Supplement: Supplementary file 1 [file jcm-13-01945-s001.zip › Supplementary Table S1.pdf]

Supplementary Table S1. Baseline characteristics of study subjects aged 20 to 75 years (N=5,229).

Characteristics	Data
Age, year (Median, IQR)	55.3 (43.0 to 64.8)
Age group (n, %)	
20 to 29 years	351 (6.7)
30 to 39 years	701 (13.4)
40 to 49 years	907 (17.3)
50 to 59 years	1,200 (22.9)
60 to 69 years	1,482 (28.3)
70 to 75 years	588 (11.2)
Sex (n, %)	
Men	3051 (58.3)
Women	2178 (41.7)
Serum Cr (mg/dL, median, IQR)	0.93 (0.76 to 1.15)
Serum Cys-C (mg/L, median, IQR)	0.92 (0.78 to 1.13)
eGFR using 2006 MDRD (mL/min/1.73 m^2, median, IQR)	77.1 (62.4 to 92.4)
eGFR using 2009 CKD-EPI Cr (mL/min/1.73 m^2, median, IQR)	84.7 (66.9 to 98.3)
eGFR using 2012 CKD-EPI Cys-C (mL/min/1.73 m^2, median, IQR)	87.3 (64.9 to 106.0)
eGFR using 2012 CKD-EPI Cr & Cys-C (mL/min/1.73 m^2, median, IQR)	86.0 (66.7 to 102.1)
eGFR using 2021 CKD-EPI Cr (mL/min/1.73 m^2, median, IQR)	88.9 (70.5 to 102.1)
eGFR using 2021 CKD-EPI Cr & Cys-C (mL/min/1.73 m^2, median, IQR)	92.4 (71.9 to 109.1)
eGFR using 2021 EKFC (mL/min/1.73 m^2, median, IQR)	80.5 (64.3 to 94.2)
Decreased eGFR (< 60 mL/min/1.73 m^2, CKD ≥ G3a)	
Based on 2006 MDRD 2006 (n, %)	1,119 (21.4%)
Based on 2009 CKD-EPI Cr (n, %)	926 (17.7%)
Based on 2012 CKD-EPI Cys-C (n, %)	1,091 (20.9%)
Based on 2012 CKD-EPI Cr & Cys-C (n, %)	983 (18.8%)
Based on 2021 CKD-EPI Cr (n, %)	785 (15.0%)
Based on 2021 CKD-EPI Cr & Cys-C (n, %)	817 (15.6%)
Based on 2021 EKFC (n, %)	1,052 (20.1%)

Abbreviations: Cr, creatinine; Cys-C, cystatin C; eGFR, estimated glomerular filtration rate.
