# Supplementary figures and images for: Comparative Analysis of Seven Equations for Estimated Glomerular Filtration Rate and Their Impact on Chronic Kidney Disease Categorization in Korean Patients at Local Clinics and Hospitals
Source: J Clin Med. 2024 Mar 27;13(7):1945. doi: 10.3390/jcm13071945 (PMC11012467; doi:10.3390/jcm13071945)

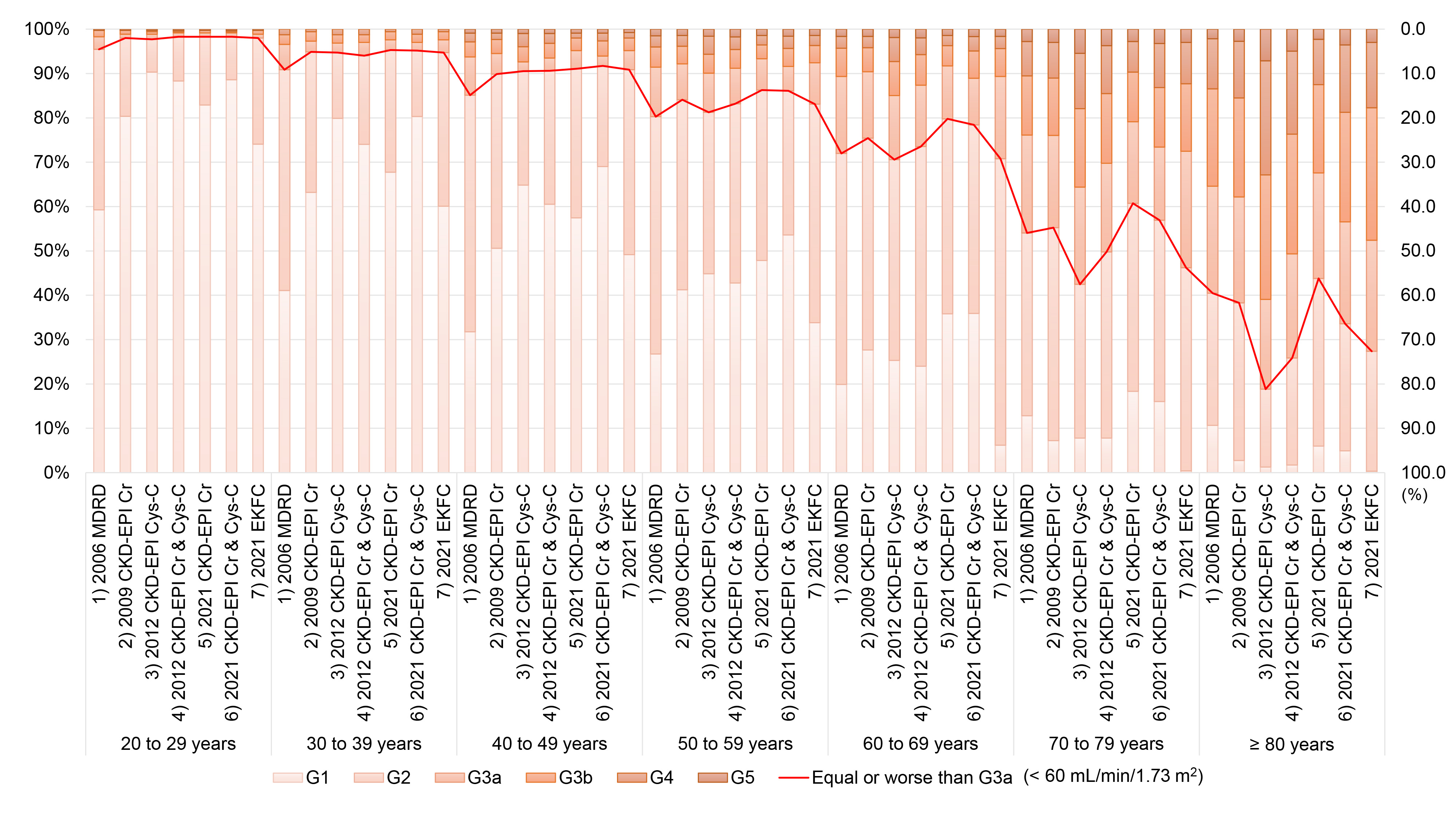

Supplement: Supplementary file 1 [file jcm-13-01945-s001.zip › Supplementary Figure S1.jpg]

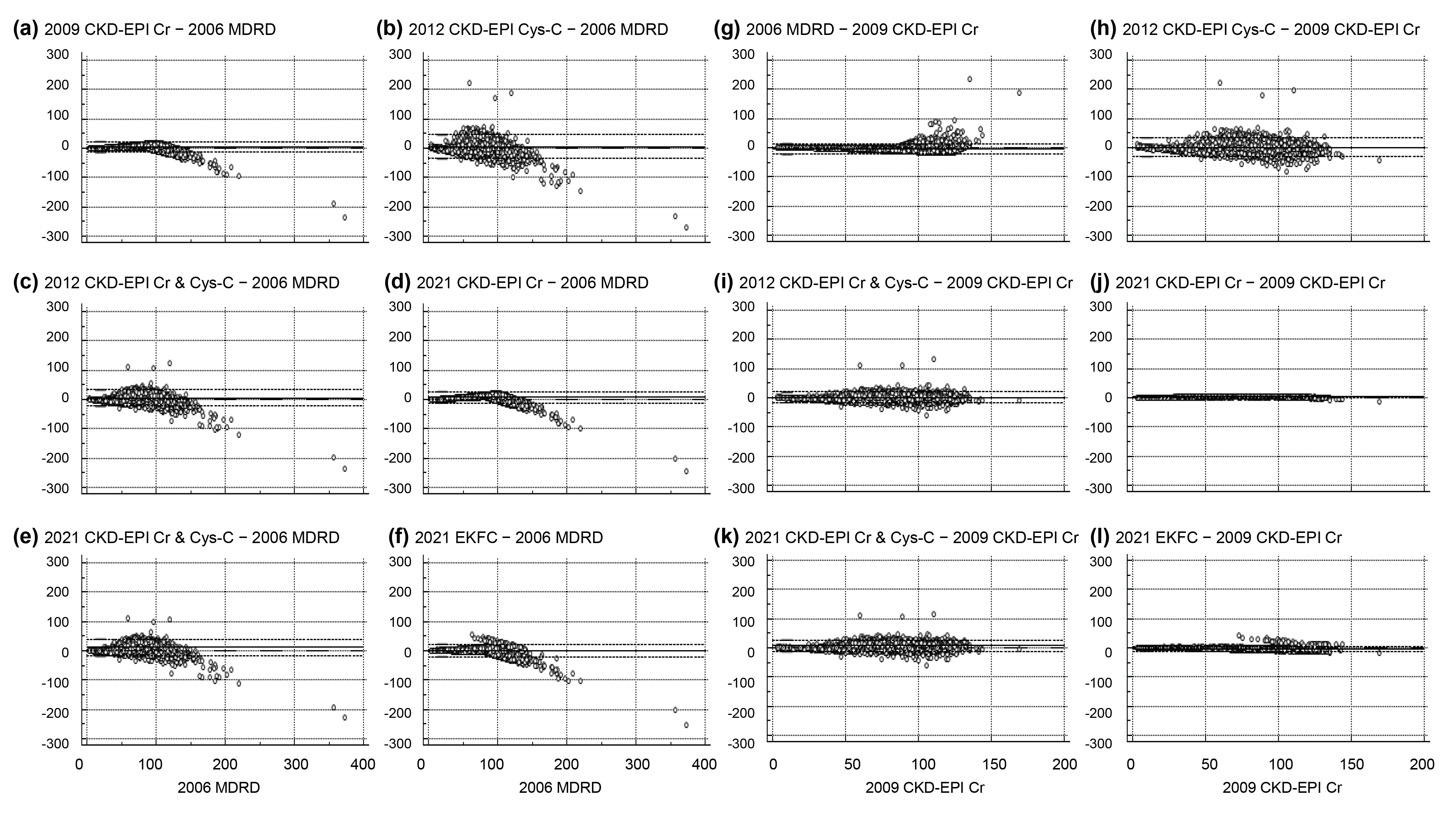

Supplement: Supplementary file 1 [file jcm-13-01945-s001.zip › Supplementary Figure S2.jpg]

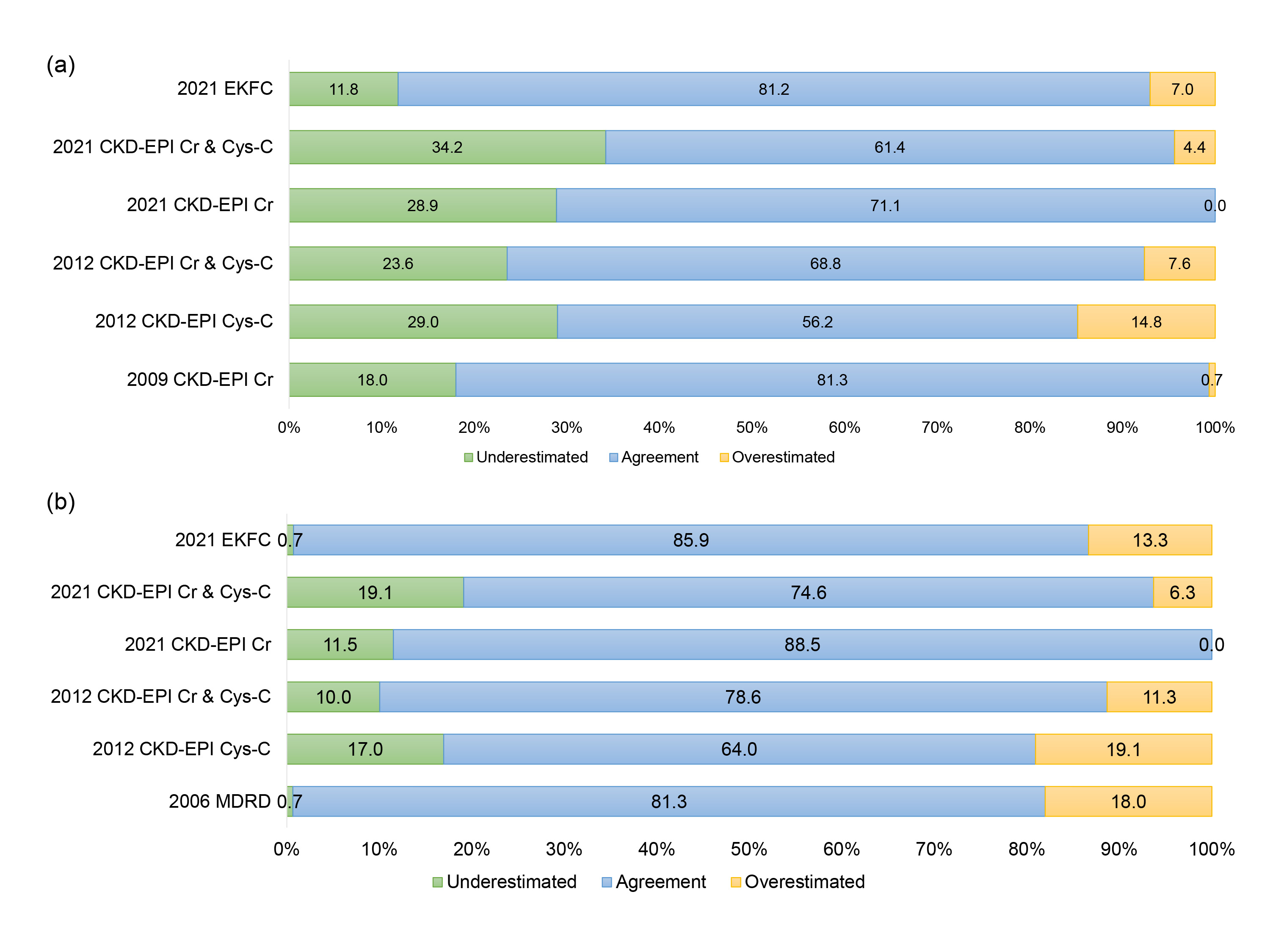

Supplement: Supplementary file 1 [file jcm-13-01945-s001.zip › Supplementary Figure S3.jpg]
